# Supplementary material for: Empirical methods for controlling false positives and estimating confidence in ChIP-Seq peaks
Source: BMC Bioinformatics. 2008 Dec 5;9:523. doi: 10.1186/1471-2105-9-523 (PMC2628906; doi:10.1186/1471-2105-9-523)
Supplement: Additional File 1 — A variety of html documents from the USeq web site detailing the available applications, their best usage, output file type descriptions, command line menus, etc. [file 1471-2105-9-523-S1.zip › USeqUserGuides/JavaDoc.zip!/JavaDoc/trans/roc/RocWindowScanner.html]

RocWindowScanner


|  |  |  |
| --- | --- | --- |
| **Overview** \| **Package** \| **Class** \| **Use** \| **Tree** \| **Deprecated** \| **Index** \| **Help** | |  |
| **PREV CLASS**   **NEXT CLASS** | **FRAMES**    **NO FRAMES**     **All Classes** |  |
| SUMMARY: NESTED \| FIELD \| CONSTR \| METHOD | DETAIL: FIELD \| CONSTR \| METHOD |  |


---


## trans.roc Class RocWindowScanner

```
java.lang.Object
  trans.roc.RocWindowScanner
```

---

``` public class RocWindowScanner extends java.lang.Object ```

Scans a window array over a range of thresholds and intersects with a list of positives to
generate the false positive rate, true positive rate, and false discovery rates.

---

**Constructor Summary**

`RocWindowScanner(java.lang.String[] args)`


| **Method Summary** | |
| --- | --- |
| `double` | `calcFDR(double threshold)`             Scans an array of Window intersecting with positives to calculate an FDR at a given threshold. |
| `void` | `calculateTotalWindowIntersectionStats()`             Calculate total # windows that intersect pos regions, no counting, ie one window overlaps two pos regions is counted once. |
| `double[]` | `findStartStopThresholds(double startThreshold, double stopThreshold)` |
| `double` | `findTargetFDRThreshold(double fdrTarget, int maxCycles)`             Attempts to find a threshold that gives the target FDR, will run through maxCycles and return closest threshold if targetFDR is not reached. |
| `static void` | `main(java.lang.String[] args)` |
| `static void` | `printDocs()` |
| `void` | `processArgs(java.lang.String[] args)`             This method will process each argument and assign new varibles |
| `void` | `removeMaskedRegions()`             Remove windows that intersect the masked regions. |

**Methods inherited from class java.lang.Object**

`equals, getClass, hashCode, notify, notifyAll, toString, wait, wait, wait`

**Constructor Detail**

### RocWindowScanner

```
public RocWindowScanner(java.lang.String[] args)
```


**Method Detail**

### main

```
public static void main(java.lang.String[] args)
```

---


### calculateTotalWindowIntersectionStats

```
public void calculateTotalWindowIntersectionStats()
```

:   Calculate total # windows that intersect pos regions, no counting, ie one window overlaps two pos regions is counted once.
    Also strip out windows that partially overlap a positive region but don't meet the fractionAcceptibleCoverage.

---


### removeMaskedRegions

```
public void removeMaskedRegions()
```

:   Remove windows that intersect the masked regions.

---


### calcFDR

```
public double calcFDR(double threshold)
```

:   Scans an array of Window intersecting with positives to calculate an FDR at a given threshold.

---


### findTargetFDRThreshold

```
public double findTargetFDRThreshold(double fdrTarget,
                                     int maxCycles)
```

:   Attempts to find a threshold that gives the target FDR, will run through maxCycles and return closest threshold if targetFDR is not reached.

---


### findStartStopThresholds

```
public double[] findStartStopThresholds(double startThreshold,
                                        double stopThreshold)
```

---


### processArgs

```
public void processArgs(java.lang.String[] args)
```

:   This method will process each argument and assign new varibles

---


### printDocs

```
public static void printDocs()
```


---


|  |  |  |
| --- | --- | --- |
| **Overview** \| **Package** \| **Class** \| **Use** \| **Tree** \| **Deprecated** \| **Index** \| **Help** | |  |
| **PREV CLASS**   **NEXT CLASS** | **FRAMES**    **NO FRAMES**     **All Classes** |  |
| SUMMARY: NESTED \| FIELD \| CONSTR \| METHOD | DETAIL: FIELD \| CONSTR \| METHOD |  |


---
